# False Dichotomies and Health Policy Research Designs: Randomized Trials Are Not Always the Answer

**DOI:** 10.1007/s11606-016-3841-9

**Published:** 2016-10-18

**Authors:** Stephen B. Soumerai, Rachel Ceccarelli, Ross Koppel

**Affiliations:** 1 Harvard Medical School Department of Population Medicine, Harvard Pilgrim Healthcare Institute, Boston, MA USA; 2 Sociology Department & LDI Wharton & School of Medicine, University of Pennsylvania, Philadelphia, PA USA

**Keywords:** research design, health interventions, quasi-experimental design, randomization

## Abstract

Some medical scientists argue that only data from randomized controlled trials (RCTs) are trustworthy. They claim data from natural experiments and administrative data sets are always spurious and cannot be used to evaluate health policies and other population-wide phenomena in the real world. While many acknowledge biases caused by poor study designs, in this article we argue that several valid designs using administrative data can produce strong findings, particularly the interrupted time series (ITS) design. Many policy studies neither permit nor require an RCT for cause-and-effect inference. Framing our arguments using Campbell and Stanley’s classic research design monograph, we show that several “quasi-experimental” designs, especially interrupted time series (ITS), can estimate valid effects (or non-effects) of health interventions and policies as diverse as public insurance coverage, speed limits, hospital safety programs, drug abuse regulation and withdrawal of drugs from the market. We further note the recent rapid uptake of ITS and argue for expanded training in quasi-experimental designs in medical and graduate schools and in post-doctoral curricula.

Information in administrative data sets is spurious by default.

John Ioannidis^1^

This statement prolongs the polarizing debate on the trustworthiness and reproducibility of findings from “available data.”^2^^–^ 4 We disagree that observational data are *always* spurious.^5^ While many weak observational studies are biased,^6^ many valid designs using administrative data produce trustworthy findings. Moreover, RCTs can be infeasible, invalid or not generalizable despite being the “gold standard.” Study end points are manipulated, or patients may not be blind to their treatment, resulting in placebo effects or exaggerated beliefs in the study treatment. Furthermore, RCTs are only useful for a fraction of health interventions, such as drugs and medical technologies.^7^^–^ 9 In addition to national policies, real-life events create other unparalleled research opportunities, e.g., government seatbelt laws, banishing certain drugs from the market, changing highway speed limits,^10^ high deductible health insurance,^11^ changes or extreme spikes in the cost of drugs,^12^^,^ 13 antibiotic controls,^14^ health outcomes of the UK’s pay-for-performance program,^15^ anti-indoor smoking regulations,^5^ and outcomes of state regulation of psychoactive drug use.^16^ These policies produced important health effects, including changes in mortality, that cannot be studied experimentally.

## THE INNOVATION Of QUASI-EXPERIMENTATION

In 1963, Campbell and Stanley, published their landmark text, “Experimental and Quasi-Experimental Designs for Research,”^17^ revised thereafter in 1979 and 2002.^5^^,^ 18 They showed several quasi-experimental research designs were often resistant to the main threats to validity such as secular trends or history bias (e.g., pre-intervention improvements in acute MI care), selection bias (e.g., study groups already healthier than controls), etc.^17^ This and other texts on quasi-experimental designs have expanded the acceptance of non-experimental studies.^5^^,^ 17 ^–^ 19

Campbell and Stanley described three main categories of research design:

1. **Randomized Experiments**: These “gold-standard” designs randomly allocate patients or clusters (e.g., health centers) to intervention and control groups. Assuming an adequate sample size, randomization addresses most sources of selection bias and confounding. However, randomized trials can still mislead if they are too small, non-representative or not really double blind.
2. **Strong quasi-experiments**: These designs compare changes in outcomes before and after a study intervention with changes in a comparable control group. Variations include: (1) comparisons of changes in hospitalization rates after a drug safety program with simultaneous changes in multiple control groups^20^ and (2) interrupted time series with or without control group(s) that measure abrupt changes from baseline trends (e.g., sudden increases in the level or slope of emergency room admissions among the chronically mentally ill soon after a cap on public insurance benefits).^21^
3. **Weak “pre-experiments**,”^17^: This group of untrustworthy studies is not included in Cochrane systematic evidence reviews of changes in health policies or programs,^22^ e.g., single observations before and after an intervention without any controls or simple cross-sectional designs that merely correlate having an intervention with mortality at a single point in time.^23^^–^ 25 These study designs cannot distinguish intervention effects from what would have occurred in the absence of the intervention [e.g., they do not address the reality that more profitable and prestigious hospitals are more likely than others to invest the vast sums required for electronic health records (EHRs)]. Such studies have influenced policymakers to spend trillions of dollars on health IT technologies with few demonstrated health benefits.^26^^–^ 28

Table 1 provides a simple hierarchy of common strong and weak designs.^6^^,^ 29

**Table 1:**
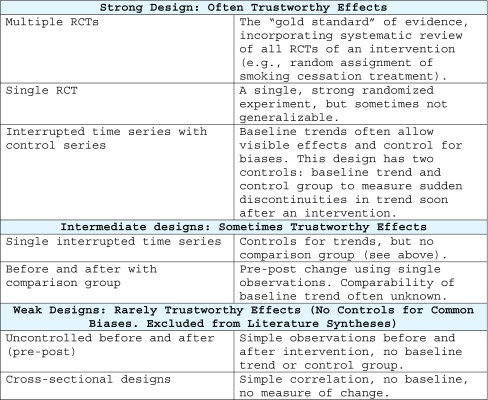
Hierarchy of Strong and Weak Designs, Based on Capacity to Control for Biases

## INTERRUPTED TIME SERIES (WITH OR WITHOUT A CONTROL GROUP): EXAMPLES OF A QUASI-EXPERIMENTAL DESIGN

Interrupted time series designs allow researchers to control for baseline secular trends, observe a sudden effect of an intervention (a change in level or slope) and assess the stability of the change over time.^30^ The design is strongest when researchers can follow another group of patients who have not experienced the intervention, i.e., a control or “comparison series.” Accessible descriptions of ITS methods are numerous.^7^^,^ 30 ^–^ 32

**Figure 1:**
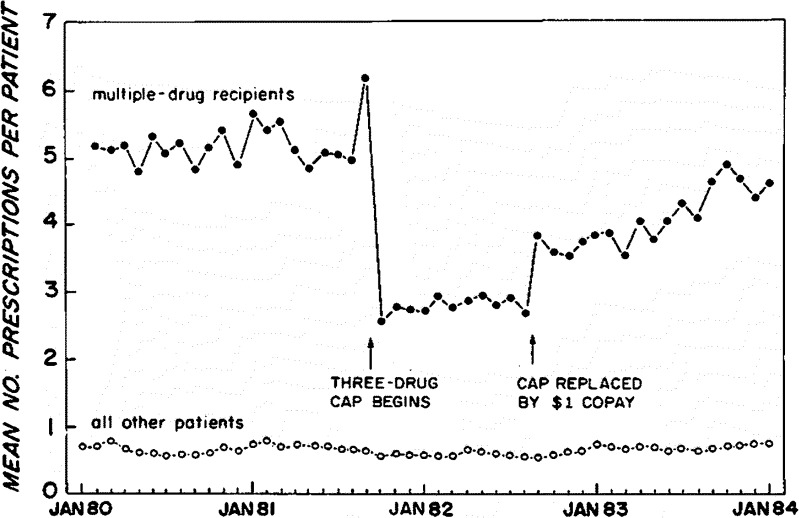
Times series effects of changes in drug benefit limits and cost sharing on the average number of constant-size prescriptions per continuously eligible patient per month among noninstitutionalized New Hampshire patients receiving multiple drugs (n = 860) and other outpatients (n = 8002)^33^

Even without a perfect comparison group, ITS can be causally persuasive. Figure 1 below shows the effect of a sudden state-imposed Medicaid three-drug reimbursement limit that restricted medications among chronically ill poor patients with cardiac and other chronic illnesses.^33^ Medication use plummeted immediately by half.

When advocacy organizations sued for damages, the state suddenly replaced the regulation with a less draconian $1 copayment per prescription after about a year. Immediately, the slope of prescription use increased to just below pre-cap levels. The off-on-off design and immediate, marked changes in the levels and slopes of the trend over 48 monthly observations do not allow or require an RCT to infer cause and effect. The graph of the longitudinal data is “worth a thousand p-values”. Government documents also reveal no “co-interventions” (simultaneous policies that could cause the outcome) and threaten the validity of such ITS designs.

Even more important to policy and economic analysis, later time-series studies visibly showed that the sudden loss of medication access substantially increased institutionalization of frail elders and increased acute mental health care use among the severely mentally ill. The cost of hospitalization and nursing home admissions dwarfed the drug savings.^21^^,^ 34 Indeed, the clearly observable ITS findings strongly contributed to many health policy improvements in the US and other countries, including rejections by many states of strict limits on drug coverage for vulnerable populations, expansion of state-funded pharmacy assistance programs,^35^ and the establishment of subsidies to drug coverage under Medicare Part D.^36^

**Figure 2:**
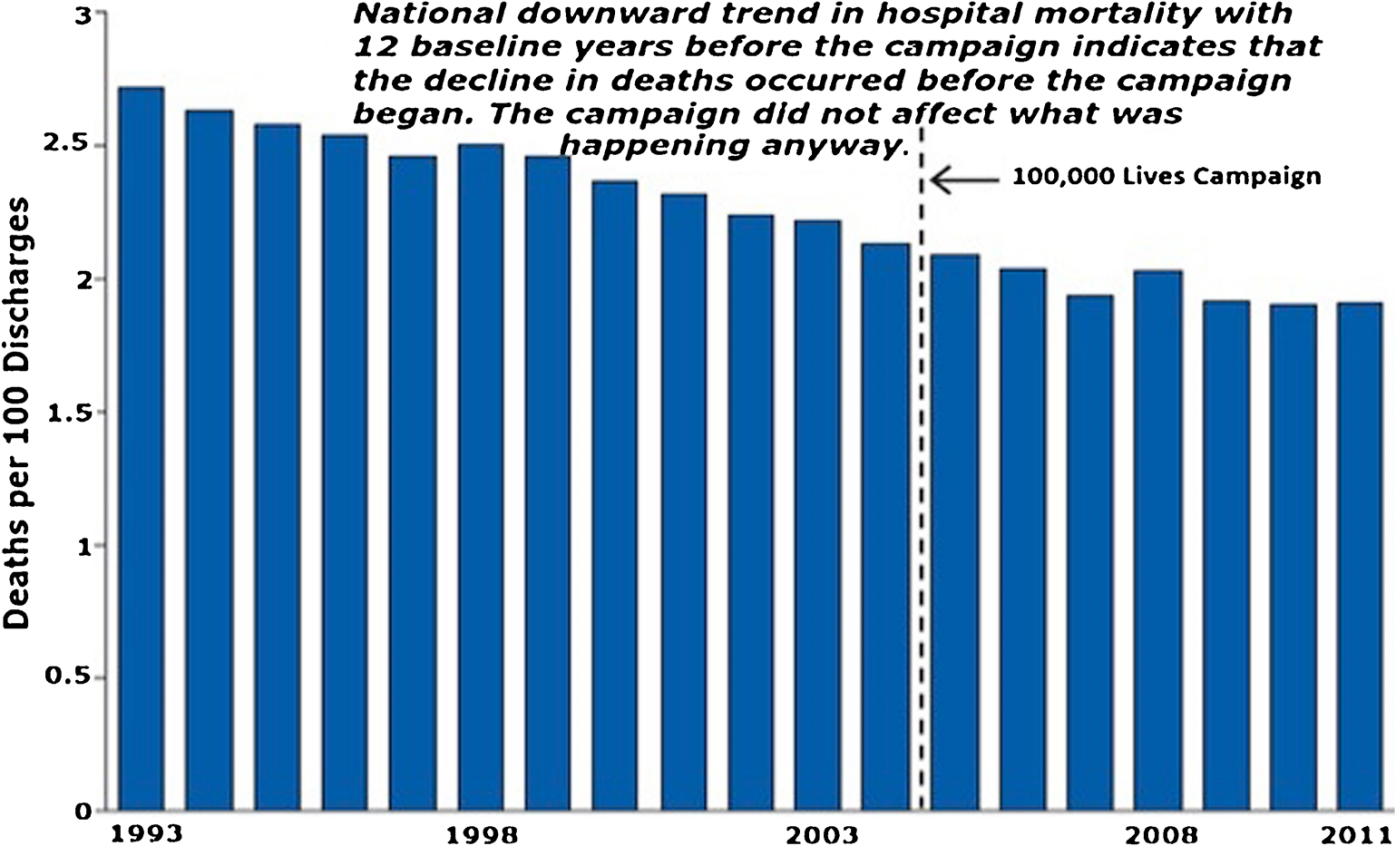
Example of a strong time-series design that controlled for history bias in the Institute for Healthcare Improvement’s (IHI) 100,000 lives campaign. Exhibit is based on data from the Agency for Healthcare Research and Quality (HCUP, 2015).^6^

ITS can also *debunk* claimed or false “effects” via elegant and parsimonious illustrations. Figure 2 demonstrates that hospital mortality was not really affected by the nationwide (US) hospital safety program of the Institute for Healthcare Improvement. The reported mortality decrease appears to evaporate when examined in relation to the ongoing secular trend: a fancy way of saying the investigators did not control for baseline decreases in mortality (history bias) and only focused on post-intervention data.^6^ No statistics are needed to seriously question the claims of 122,000 lives saved. Using only administrative data without a control group, it is clear the decline was already happening.

**Figure 3:**
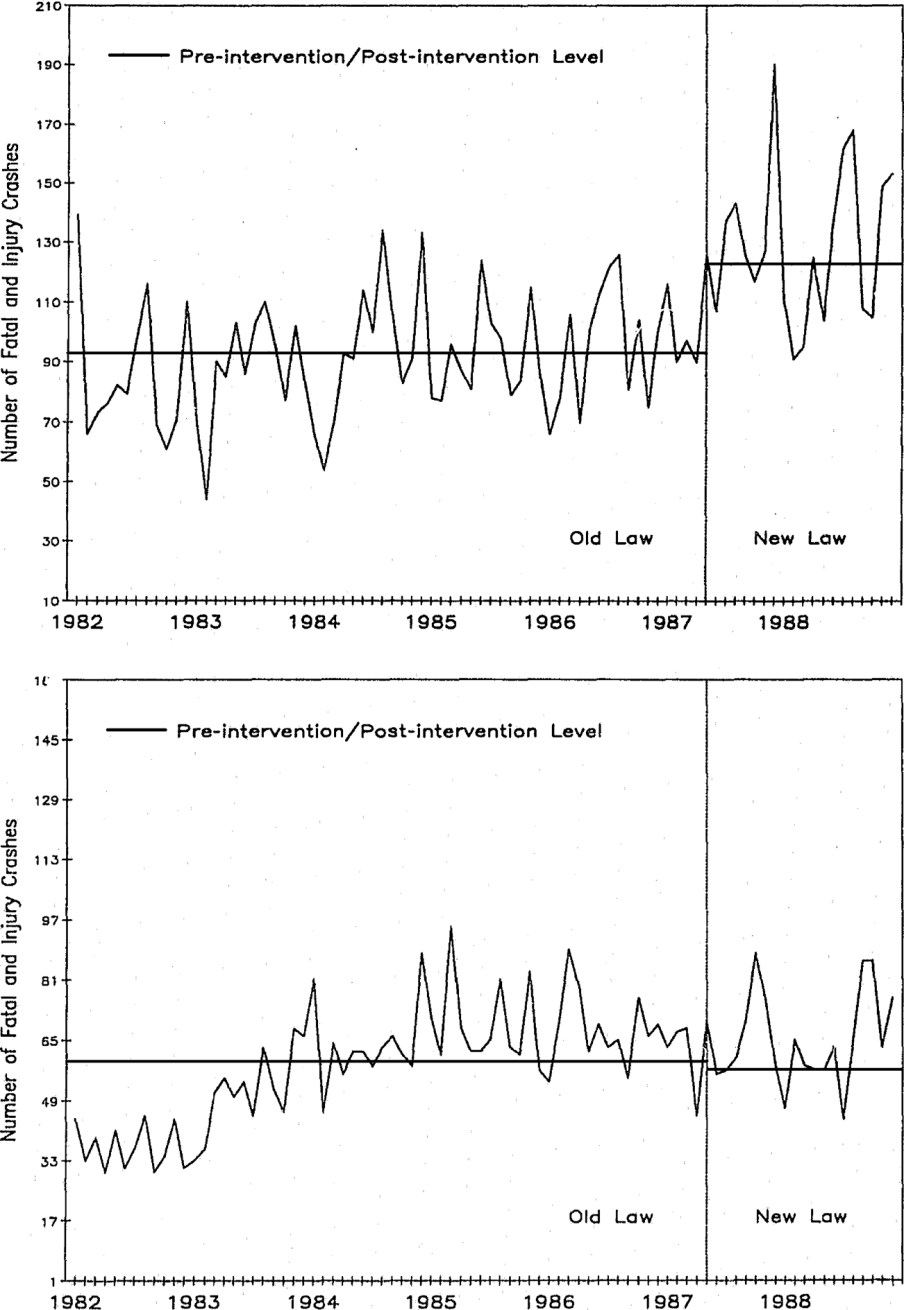
Upper graph shows fatal and injurious crashes on Arizona interstate highways with the increase to 65 MPH maximum speed limit. The lower graph indicates fatal and injurious crashes on Arizona interstate highways with no change in the 55 MPH maximum speed limit^10^

Figure 3 shows increased fatal and injurious car crashes on Arizona highways with a new 65 MPH vs. a previous 55 MPH speed limit. It is an especially powerful example of ITS because the study group data come from only those highways with posted higher speed limits reflecting the new law. The large and marked upward shift immediately after the change in speed limit is obvious. In fact, Fig. 3 also displays fatal and injurious car crashes on AZ highways that did not increase the posted speed limits. In this graph there is no sudden shift in fatal and injurious car crashes. No RCT would be feasible in such a study, and the ITS and control group provide strong data on the impact of this new law.

Often the most powerful evidence is a graph that simply and reliably shows the trend and the effects of an intervention. While not infallible, ITS designs can often supplement, replicate or replace some RCTs.^7^

## DISCUSSION

Between 1996 and 2015, the number of studies in PubMed identified as “interrupted time series” increased from 12 to 239 per year. Even this jump substantially undercounts such studies because many are described simply as “time series.”

We hope the increasing use of this common and useful design is accompanied by an expanding acceptance of other strong non-experimental designs by medical journals and scholars.^5^ As teachers we have an obligation to explain quasi-experiments to future medical researchers, along with the difference between strong and weak research designs in evaluating system-wide innovations affecting health. RCTs can only address a small proportion of interventions affecting the cost, quality and outcomes of medical and health policy interventions.

Given the influence research can have on policy, it is distressing that so much research is untrustworthy because of faulty research designs. This unease is the subject of a recent article in the US Centers for Disease Control’s *Preventing Chronic Disease* entitled, “*How do you Know Which Health Care Effectiveness Research You Can Trust? A Guide to Study Design for the Perplexed*”^6^ http://www.cdc.gov/pcd/issues/2015/15_0187.htm. Similarly, the National Institutes of Health (NIH) are deeply concerned about the phenomenon of “the non-reproducibility of research.”^37^

Research design is often missing in the medical curriculum. Poorly controlled studies are the rule, not the exception.^38^ This confuses the public, policymakers, media and researchers themselves. The countless reports (and reversals of findings)^39^ regarding micronutrients and physical activities that grossly exaggerate lives saved is a case in point.^39^ Accompanying the increase in what is viewed as flip-flopping research, we see a marked rise in media and researcher websites devoted to uncovering what is viewed as biased or fraudulent research.

Research design may well be the first consideration in addressing the trustworthiness of research findings.^6^ Medical and graduate school curricula should emphasize the weaknesses of uncontrolled or cross-sectional designs and should include both experimental and strong quasi-experimental designs. Well-controlled and -designed studies can save lives,^40^ while biased ones promote inefficient expenditures for useless programs, cause patient safety dangers and suffering, and jeopardize public health.^6^
